# Acylphenols and Dimeric Acylphenols from the Genus *Myristica*: A Review of Their Phytochemistry and Pharmacology

**DOI:** 10.3390/plants12081589

**Published:** 2023-04-09

**Authors:** Muhamad Aqmal Othman, Yasodha Sivasothy

**Affiliations:** 1 Department of Chemistry, Faculty of Science, University of Malaya, Kuala Lumpur 50603, Malaysia; 2 Centre for Natural Products Research and Drug Discovery (CENAR), University of Malaya, Kuala Lumpur 50603, Malaysia; 3 School of Pharmacy, Monash University Malaysia, Jalan Lagoon Selatan, Bandar Sunway 47500, Malaysia

**Keywords:** Myristicaceae, *Myristica*, acylphenols, dimeric acylphenols

## Abstract

The genus *Myristica* is a medicinally important genus belonging to the Myristicaceae. Traditional medicinal systems in Asia have employed plants from the genus *Myristica* to treat a variety of ailments. Acylphenols and dimeric acylphenols are a rare group of secondary metabolites, which, to date, have only been identified in the Myristicaceae, in particular, in the genus *Myristica*. The aim of the review would be to provide scientific evidence that the medicinal properties of the genus *Myristica* could be attributed to the acylphenols and dimeric acylphenols present in the various parts of its plants and highlight the potential in the development of the acylphenols and dimeric acylphenols as pharmaceutical products. SciFinder-n, Web of Science, Scopus, ScienceDirect, and PubMed were used to conduct the literature search between 2013–2022 on the phytochemistry and the pharmacology of acylphenols and dimeric acylphenols from the genus *Myristica*. The review discusses the distribution of the 25 acylphenols and dimeric acylphenols within the genus *Myristica*, their extraction, isolation, and characterization from the respective *Myristica* species, the structural similarities and differences within each group and between the different groups of the acylphenols and dimeric acylphenols, and their in vitro pharmacological activities.

## 1. Introduction

The genus *Myristica*, one of the most common genera within the Myristicaceae, consists of 120 species distributed in Australia, South Asia, from west Polynesia, Oceania, and East India to the Philippines [1,2,3]. The trees are of various sizes, reaching up to about 120 feet in height, with buttresses or stilt roots. The bark is either black or brown in color, brittle, and scaly or fissured. The leaves are alternate, generally long, leathery, sometimes hairy, dark shiny green above and glaucous below. The inflorescences are branching or axillary panicles. The flowers are mostly tiny, sometimes sweetly scented, flask or bell shaped, white or pale yellow in color, with brown hair on the outer side and glabrous on the inner side with three acute reflexed lobes. The fruits are usually large, round-to-oblong usually longer than broad, pointed, yellow, or red upon ripening, sometimes hairy, have a thick fleshy wall, ultimately, splitting into two halves to expose the single large hard seed (nutmeg) [4]. The seed is covered in a pink or red waxy flesh that resembles a lace-like membrane, known as the mace [5].

Nutmeg and mace from certain members of the genus *Myristica,* for example, *M. fragrans* Houtt, *M. malabarica* Lam., and *M. argentea* Warb., are spices with similar tastes. Nutmeg is sweeter, while mace is known to release more delicate flavors [5]. Nutmeg and mace are added during cooking to enhance the flavor and aroma of savory dishes (e.g., potato dishes, sauces, curries), desserts (e.g., cakes, muffins, pies), and beverages (e.g., tea, mulled wine). Mace at times is preferred in some dishes for the orange color (saffron-like color) that it imparts to the food. Nutmeg is also used as an ingredient in some curry powders [5,6,7,8]. 

The plants of the genus *Myristica*, in particular, the fruits and, to a lesser extent, the leaves and bark, have been extensively used in folk medicine in Asia to treat many ailments such as rheumatism, muscle spasm, decreased appetite, jaundice, skin diseases, anxiety, depression, coughs, bronchitis, asthma, fever, burning sensations, kidney disorders, liver disorders, to improve digestion, to promote wound healing, and to manage GI conditions such as colic, nausea, diarrhea, and flatulence [6,7,9,10,11,12,13,14,15]. 

Phytochemical investigation of the genus *Myristica* has led to the isolation and characterization of various classes of phenolic type secondary metabolites such as acylphenols, dimeric acylphenols, flavonoids, lignans, and neolignans [5,8,15,16,17,18,19,20,21,22,23,24,24,25,26,27,28,29,30,31,32,33]. These phenolic type secondary metabolites have been reported to exhibit numerous pharmacological activities namely cytotoxic activity, nitric oxide inhibitory activity, AMPK activators, anticariogenic activity, LDL antioxidant activity, DPPH free radical scavenging activity, lipid peroxidation activity, anti-quorum sensing activity, acetylcholinesterase inhibitory activity, anti-inflammatory activity, anti-platelet activity, antifungal activity, and COX-2 inhibitory activity [5,8,15,16,18,19,22,23,24,25,26,30,31,32,33,34].

The present review aims to provide an insight into the therapeutic potential of the genus *Myristica* being used as a source of bioactive phenolic type secondary metabolites with the emphasis being on the acylphenols and dimeric acylphenols that can be further developed into pharmaceutical products. Thus far, there has been no such study on this medicinally important genus. This study, which reports on the pharmacological activities of the acylphenols and dimeric acylphenols, will serve as a chemical database for future research while providing researchers with a framework for potential future studies.

## 2. Methodology

The review was conducted using five electronic databases: SciFinder-n, Web of Science, Scopus, ScienceDirect, and PubMed. The scientific articles obtained were refined by document type (full length articles, short communications, notes, and letters), language (English), and publication year (2013–2022). Literature reviews, systematic reviews, meta-analysis, conference proceedings, and patents were excluded from this review. 

The main topic of the search was “*Myristica*”, and the search was refined using the following keywords in both their singular and plural forms: “acylphenol” OR “acylphenols”, “dimeric acylphenol” OR dimeric acylphenols”, “malabaricone” OR “malabaricones”, “giganteone” OR “giganteones”, “maingayone” OR “maingayones”, “promalabaricone” OR “promalabaricones”. 

## 3. Results

**Figure 1:**
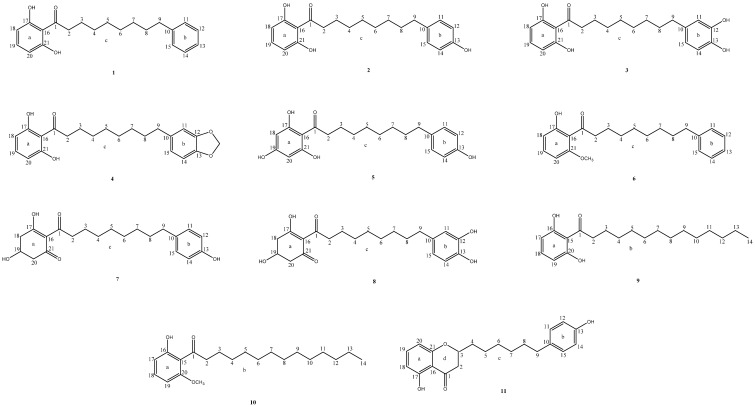

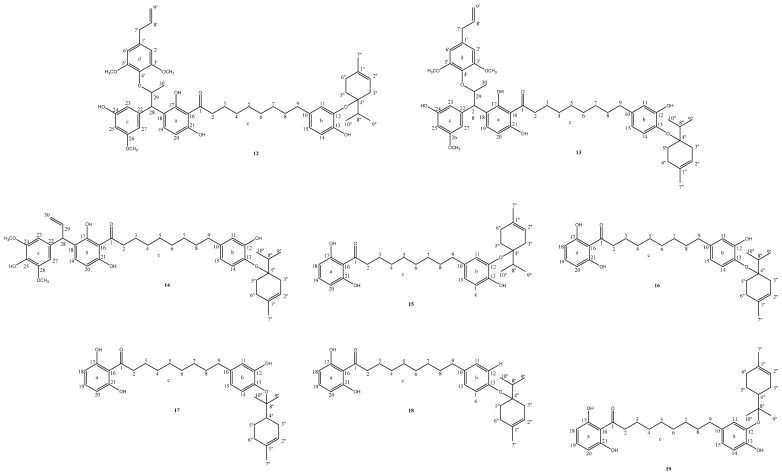
Acylphenols from the genus *Myristica*.

**Figure 2:**
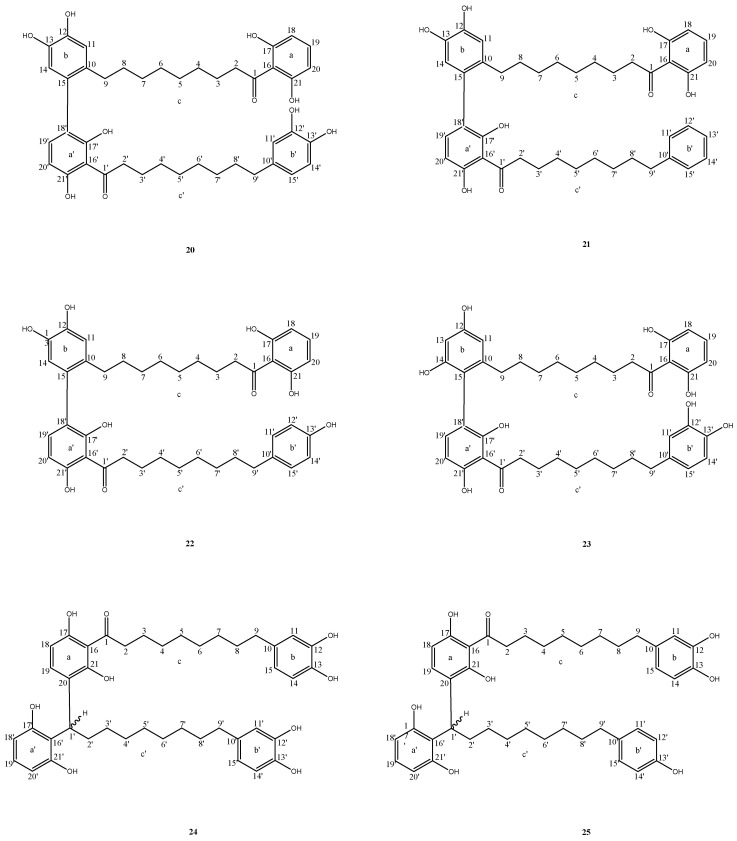
Dimeric Acylphenols from the genus *Myristica*.

The initial SciFinder-n, Web of Science, Scopus, ScienceDirect, and PubMed searches using the search term “*Myristica*”, which was refined by document type, language, and publication year, yielded 999, 919, 650, 587, and 540 potentially relevant scientific articles, respectively. After further refining the respective searches using a specific set of keywords and after excluding multiple entries, a total of 35 scientific articles remained. These 35 scientific articles specifically focused on the phytochemistry and the pharmacology of the acylphenols and dimeric acylphenols from the genus *Myristica* and, therefore, were considered relevant for inclusion in this review. The following are the number of scientific articles obtained for each year between 2013–2022; 2013 (*n* = 1), 2014 (*n* = 4), 2015 (*n* = 2), 2016 (*n* = 7), 2017 (*n* = 2), 2018 (*n* = 3), 2019 (*n* = 2), 2020 (*n* = 4), 2021 (*n* = 3), and 2022 (*n* = 7). Table 1, Table 2, Table 3, Table 4, Table 5, Table 6, Table 7, Table 8, Table 9, Table 10, Table 11, Table 12 and Table 13 and Figure 1 and Figure 2 summarize the main findings obtained from our analyses of the 35 scientific articles.

Scientific articles reporting only on the pharmacological activities of the crude extract(s) were excluded from this review. Scientific articles reporting only on the pharmacological activities of the fractionated extract(s), which have been identified to contain either acylphenols or dimeric acylphenols, were also excluded from this review. 

**Table 1:** Distribution of acylphenols and dimeric acylphenols within the genus *Myristica*.

*Myristica* spp.	Part of the Species Investigated	Acylphenols	Dimeric Acylphenols	Reference
*Myristica beddomei* subsp. *sphaerocarpa* W.J. de Wilde	Rind Seeds Bark	Malabaricone A (**1**) Malabaricone B (**2**) Malabaricone C (**3**) Malabaricone D (**4**) Promalabaricone B (**7**) 1-(2,6-dihydroxyphenyl)tetradecan-1-one (**9**) Malabaricone A (**1**) Malabaricone B (**2**) Malabaricone C (**3**) Malabaricone D (**4**) 1-(2,6-dihydroxyphenyl)tetradecan-1-one (**9**) Malabaricone A (**1**) Malabaricone B (**2**) 1-(2,6-dihydroxyphenyl)tetradecan-1-one (**9**)		[35]
*Myristica cinnamomea* King	Bark	Malabaricone A (**1**) Malabaricone B (**2**) Malabaricone C (**3**) Cinnamomeone A (**11**)	Giganteone A (**20**) Giganteone D (**21**)	[36,37]
*Myristica cinnamomea* King	Fruits	Malabaricone A (**1**) Malabaricone B (**2**) Malabaricone C (**3**) Malabaricone E (**5**)	Maingayone A (**24**) Maingayone B (**25**)	[38]
*Myristica fatua* Houtt.	Bark	Malabaricone B (**2**) Malabaricone C (**3**)		[39]
*Myristica fatua* Houtt.	Seeds	Malabaricone A (**1**) Malabaricone B (**2**) Malabaricone C (**3**) Promalabaricone B (**7**) 1-(2,6-dihydroxyphenyl)tetradecan-1-one (**9**)		[40]
*Myristica fatua* Houtt. var. *magnifica* (Bedd.) Sinclair	Bark	Malabaricone A (**1**) Malabaricone B (**2**) Malabaricone C (**3**) 1-(2-hydroxy-6-methoxyphenyl)-9-(4-hydroxyphenyl)nonan-1-one (**6**) 1-(2,6-dihydroxyphenyl)tetradecan-1-one (**9**) 1-(2-hydroxy-6-methoxyphenyl)tetradecan-1-one (**10**)		[41]
*Myristica fragrans* Houtt.	Seeds	Malabaricone C (**3**)		[42]
*Myristica fragrans* Houtt.	Seeds	Malabaricone C (**3**)		[14]
*Myristica fragrans* Houtt.	Kernel	Malabaricone B (**2**) Malabaricone C (**3**)		[12]
*Myristica fragrans* Houtt.	Aril	Malabaricone C (**3**)		[7]
*Myristica fragrans* Houtt.	Seeds	Malabaricone B (**2**) Malabaricone C (**3**)		[43]
*Myristica fragrans* Houtt.	Kernel	Malabaricone C (**3**)		[11]
*Myristica fragrans* Houtt.	Seeds	Malabaricone B (**2**) Malabaricone C (**3**)		[10]
*Myristica fragrans* Houtt.	Aril	Malabaricone C (**3**)		[44]
*Myristica fragrans* Houtt.	Fruits	Malabaricone C (**3**)		[45]
*Myristica fragrans* Houtt.	Aril	Malabaricone A (**1**) Malabaricone C (**3**)		[46]
*Myristica fragrans* Houtt.	Seeds	Malabaricone A (**1**) Myrifratin A (**12**) Myrifratin B (**13**) Myrifratin C (**14**) Myrifratin D (**15**) Myrifratin E (**16**) Myrifratin F (**17**) Myrifratin G (**18**) (−)-1-(2,6-dihydrox- yphenyl)-9-[4-hydroxy-3-(p-menth-1-en-8-oxy)-phenyl]-1-nonanone (**19**)		[47]
*Myristica malabarica* Lam.	Rind	Malabaricone A (**1**) Malabaricone B (**2**) Malabaricone C (**3**) Malabaricone D (**4**)		[13,48]
*Myristica malabarica* Lam.	Rind	Malabaricone A (**1**) Malabaricone B (**2**) Malabaricone C (**3**) Malabaricone D (**4**)		[49,50,51]
*Myristica malabarica* Lam.	Rind	Malabaricone C (**3**)		[52]
*Myristica malabarica* Lam.	Rind	Malabaricone C (**3**)		[53]
*Myristica malabarica* Lam.	Seeds	Malabaricone C (**3**)		[54]
*Myristica malabarica* Lam.	Rind	Malabaricone A (**1**) Malabaricone B (**2**) Malabaricone C (**3**) Malabaricone D (**4**) Promalabaricone B (**7**) Promalabaricone C (**8**) 1-(2,6-dihydroxyphenyl)tetradecan-1-one (**9**)		[55]
*Myristica maxima* Warb.	Bark	Malabaricone A (**1**) Malabaricone B (**2**) Malabaricone C (**3**)	Giganteone A (**20**) Giganteone C (**22**) Giganteone E (**23**) Maingayone A (**24**) Maingayone B (**25**)	[56]
*Myristica philippensis* Lam.	Leaves	Malabaricone B (**2**) Malabaricone C (**3**)		[57]

## 4. Discussion

### 4.1. Phytochemical Investigation

The chemical structures of the acylphenols and dimeric acylphenols and their names in relation to the corresponding *Myristica* species are illustrated in Figure 1 and Figure 2 and presented in Table 1, respectively. Table 2 summarizes the techniques which were used to extract, isolate, and characterize the acylphenols and dimeric acylphenols from the respective *Myristica* species. 

**Table 2:** Extraction, isolation and characterization of the acylphenols and dimeric acylphenols from the genus *Myristica*.

Species	Part of the Species Investigated	Method of Extraction	Method of Isolation	Method of Characterization	Name of Acylphenols	Name of Dimeric Acylphenols	Reference
*M. beddomei* subsp. *sphaerocarpa* W.J. de Wilde	Rind Seeds Bark	Extracted sequentially with hexane, dichloromethane, acetone, ethanol, and water at room temperature. Acetone extract was subjected to further analysis. Extracted sequentially with hexane, dichloromethane, acetone, ethanol and water at room temperature. Dichloromethane extract was subjected to further analysis. Extracted sequentially with hexane, dichloromethane, acetone, ethanol, and water at room temperature. Acetone extract was subjected to further analysis.	Column chromatography (silica gel) Column chromatography (silica gel) Column chromatography (silica gel)	IR, UV, ESI/HRMS, NMR IR, UV, ESI/HRMS, NMR IR, UV, ESI/HRMS, NMR	Malabaricone A (**1**) Malabaricone B (**2**) Malabaricone C (**3**) Malabaricone D (**4**) Promalabaricone B (**7**) 1-(2,6-dihydroxyphenyl)tetradecan-1-one (**9**) Malabaricone A (**1**) Malabaricone B (**2**) Malabaricone C (**3**) Malabaricone D (**4**) 1-(2,6-dihydroxyphenyl)tetradecan-1-one (**9**) Malabaricone A (**1**) Malabaricone B (**2**) 1-(2,6-dihydroxyphenyl)tetradecan-1-one (**9**)		[35]
*M. cinnamomea* King	Bark	Extracted sequentially with hexane and acetone at room temperature. Hexane extract was subjected to further analysis.	Column chromatography (silica gel)	IR, UV, LCMS-IT-TOF, NMR	Cinnamomeone A (**11**)	Giganteone D (**21**)	[36]
*M. cinnamomea* King	Bark	Extracted sequentially with hexane and acetone at room temperature. Acetone extract was re-extracted with ethyl acetate. Ethyl acetate fraction was subjected to further analysis.	Column chromatography (silica gel)	IR, UV, LCMS-IT-TOF, NMR	Malabaricone A (**1**) Malabaricone B (**2**) Malabaricone C (**3**)	Giganteone A (**20**)	[37]
*M. cinnamomea* King	Fruits	Extracted with ethyl acetate at room temperature.	Column chromatography (silica gel/Sephadex LH 20) Preparative TLC	IR, UV, LCMS-IT-TOF, NMR	Malabaricone A (**1**) Malabaricone B (**2**) Malabaricone C (**3**) Malabaricone E (**5**)	Maingayone A (**24**) Maingayone B (**25**)	[38]
*M. fatua* Houtt.	Bark	Extracted with methanol at room temperature.	Column chromatography (silica gel)	ESIMS, NMR	Malabaricone B (**2**) Malabaricone C (**3**)		[39]
*M. fatua* Houtt.	Seeds	Extracted with dichloromethane at room temperature.	Column chromatography (silica gel)	HRESIMS, NMR	Malabaricone A (**1**) Malabaricone B (**2**) Malabaricone C (**3**) Promalabaricone B (**7**) 1-(2,6-dihydroxyphenyl)tetradecan-1-one (**9**)		[40]
*M. fatua* Houtt. var. *magnifica* (Bedd.) Sinclair	Bark	Extracted with dichloromethane at room temperature.	Column chromatography (silica gel)	IR, HRESIMS, NMR	Malabaricone A (**1**) Malabaricone B (**2**) Malabaricone C (**3**) 1-(2-hydroxy-6-methoxyphenyl)-9-(4-hydroxyphenyl)nonan-1-one (**6**) 1-(2,6-dihydroxyphenyl)tetradecan-1-one (**9**) 1-(2-hydroxy-6-methoxyphenyl)tetradecan-1-one (**10**)		[41]
*M. fragrans* Houtt.	Seeds	Refluxed with methanol. Methanolic extract was partitioned with hexane, ethyl acetate, and butanol. Ethyl acetate fraction was subjected to further analysis.	Column chromatography (silica gel) Reversed phase column chromatography (ODS-A) Semipreparative HPLC (ODS-A)	NA	Malabaricone C (**3**)		[42]
*M. fragrans* Houtt.	Kernel	Extracted with methanol at room temperature. Solid phase extraction of the methanol extract with hexane, ethyl acetate, and methanol at room temperature. Ethyl acetate extract was subjected to further analysis.	Flash MPLC (silica HP 50) Preparative HPLC	HRMS and NMR	Malabaricone B (**2**) Malabaricone C (**3**)		[12]
*M. fragrans* Houtt.	Seeds	Extracted with ethanol at room temperature. Ethanolic extract was partitioned with hexane and ethyl acetate. Hexane fraction was subjected to further analysis.	Column chromatography (silica gel/RP C-18/Sephadex LH-20) Recycling HPLC	HREIMS, NMR	Malabaricone B (**2**) Malabaricone C (**3**)		[43]
*M. fragrans* Houtt	Aril	Refluxed with methanol.	Preparative HPLC	IR, UV, ESIMS. HREIMS, NMR	Malabaricone C (**3**)		[7]
*M. fragrans* Houtt.	Kernel	Extracted with methanol at room temperature using a sonicator. Solid phase extraction of the methanol extract with hexane, ethyl acetate and methanol at room temperature. Ethyl acetate extract was subjected to further analysis.	Flash column chromatography (silica gel) Semipreparative HPLC	NA	Malabaricone C (**3**)		[11]
*M. fragrans* Houtt.	Seeds	Extracted with methanol at room temperature.	Column chromatography (silica gel) Preparative TLC	NA	Malabaricone B (**2**) Malabaricone C (**3**)		[10]
*M. fragrans* Houtt.	Aril	Extracted sequentially with hexane, dichloromethane, ethyl acetate, and methanol at room temperature using a sonicator. The ethyl acetate and methanol extracts were combined for further analysis.	Column chromatography (silica gel) Preparative TLC	ESIMS. NMR	Malabaricone C (**3**)		[44]
*M. fragrans* Houtt.	Fruits	Extracted with 50% ethanol.	Preparative HPLC	UV, HRMS, NMR	Malabaricone C (**3**)		[45]
*M. fragrans* Houtt.	Aril	Macerated with methanol: water (80:20) at room temperature, Aqueous methanolic extract was partitioned with hexane, chloroform, and ethyl acetate. Ethyl acetate extract was subjected to further analysis.	Column chromatography (silica gel/Sephadex LH-20)	MS, NMR	Malabaricone A (**1**) Malabaricone C (**3**)		[46]
*M. fragrans* Houtt.	Seeds	Percolated with 80% ethanol at room temperature. Aqueous ethanolic extract was partitioned with petroleum ether and methanol. Methanolic extract was further partitioned with ethyl acetate. Petroleum ether and ethyl acetate extracts were subjected to further analysis.	Column chromatography (silica gel/RP C-18) Semipreparative HPLC	IR, UV, HREIMS, NMR	Malabaricone A (**1**) Myrifratin A (**12**) Myrifratin B (**13**) Myrifratin C (**14**) Myrifratin D (**15**) Myrifratin E (**16**) Myrifratin F (**17**) Myrifratin G (**18**) (−)-1-(2,6-dihydrox- yphenyl)-9-[4-hydroxy-3-(p-menth-1-en-8-oxy)-phenyl]-1-nonanone (**19**)		[47]
*M. malabarica* Lam.	Rind	Extracted with methanol at room temperature.	Column chromatography (silica gel)	NMR	Malabaricone C (**3**)		[53]
*M. malabarica* Lam.	Seeds	Defatted with dichloromethane and subsequently extracted with acetone at room temperature. Acetone extract was subjected to further analysis	Column chromatography (silica gel)	MS, NMR	Malabaricone C (**3**)		[54]
*M. malabarica* Lam.	Rind	Extracted with methanol at room temperature.	Column chromatography (silica gel/Sephadex LH-20)	IR, UV, ESIMS. EIMS, NMR	Malabaricone A (**1**) Malabaricone B (**2**) Malabaricone C (**3**) Malabaricone D (**4**) Promalabaricone B (**7**) Promalabaricone C (**8**) 1-(2,6-dihydroxyphenyl)tetradecan-1-one (**9**)		[55]
*M. maxima* Warb.	Bark	Extracted with dichloromethane and ethyl acetate at room temperature. Dichloromethane extract was subjected to further analysis	Column chromatography (silica gel/Sephadex LH 20) Preparative TLC Preparative HPLC	IR, UV, LCMS-IT-TOF, NMR	Malabaricone A (**1**) Malabaricone B (**2**) Malabaricone C (**3**)	Giganteone A (**20**) Giganteone C (**22**) Giganteone E (**23**) Maingayone A (**24**) Maingayone B (**25**)	[56]
*M. philippensis* Lam.	Leaves	Extracted with dichloromethane at room temperature.	Column chromatography (silica gel)	NMR	Malabaricone B (**2**) Malabaricone C (**3**)		[57]

#### 4.1.1. Distribution of the Acylphenols and Dimeric Acylphenols within the Genus *Myristica*

A total of twenty-five acylphenols and dimeric acylphenols have been identified in eight different species within the genus *Myristica*: *M. beddomei* subsp. *sphaerocarpa* W.J. de Wilde from India, *M. cinnamomea* King from Malaysia, *M. fatua* Houtt. from either Indonesia or India, *M. fatua* Houtt. var. *magnifica* (Bedd.) Sinclair from India, *M. fragrans* Houtt. from either Vietnam, Indonesia, Taiwan, Sri Lanka or India, *M. malabarica* Lam. from India, *M. maxima* Warb. from Malaysia, and *M. philippensis* Lam. from the Philippines (Table 1). Though acylphenols were characterized in all of the eight species, it is interesting to note that the dimeric acylphenols were only reported to be present in the *Myristica* species collected from Malaysia: *M. cinnamomea* and *M. maxima*. 

#### 4.1.2. Extraction, Isolation, and Characterization of the Acylphenols and Dimeric Acylphenols

The acylphenols and dimeric acylphenols were extracted from either the fruits (rind, seeds, aril, kernel), the bark, or the leaves of the different *Myristica* species using the solvent extraction method. The extraction of the plant materials was carried out either at room temperature or under reflux using organic solvents or aqueous alcohol (Table 2). 

The resulting crude solvent extracts were either used directly or further partitioned with organic solvents and subjected to various chromatographic techniques mainly silica gel column chromatography, preparative TLC, and preparative HPLC to isolate and purify the acylphenols and dimeric acylphenols (Table 2). 

The structures of these acylphenols and dimeric acylphenols were subsequently elucidated by a combination of various spectroscopic techniques such as infrared spectroscopy (IR), ultraviolet-visible spectroscopy (UV-Vis), mass spectrometry (MS), and nuclear magnetic resonance spectroscopy (NMR) (Table 2). 

Altogether, five acylphenols were isolated and characterized from *M. beddomei* subsp. *sphaerocarpa*, five acylphenols and four dimeric acylphenols from *M. cinnamomea*, five acylphenols from *M. fatua*, six acylphenols from *M. fatua* var. *magnifica*, eleven acylphenols from *M. fragrans*, seven acylphenols from *M. malabarica*, three acylphenols and five dimeric acylphenols from *M. maxima,* and two acylphenols from *M. philippensis*.

#### 4.1.3. Structures of the Acylphenols and Dimeric Acylphenols

Among the twenty-five acylphenols and dimeric acylphenols identified through this search, nineteen were characterized as acylphenols (**1**–**19**), while the remaining six as dimeric acylphenols (**20**–**25**) (Table 1, Figure 1 and Figure 2). The following sections will discuss the structural similarities and differences within each group of the acylphenols and dimeric acylphenols and between the different groups of the acylphenols and dimeric acylphenols. 

##### Acylpenols

The acylphenols can be further classified into five types: Type 1 (**1**–**6**), Type 2 (**7** and **8**), Type 3 (**9** and **10**), Type 4 (**11**), and Type 5 (**12**–**19**).

Type 1 Acylphenols

The basic skeleton of compounds **1**–**6** was constructed from three substructures: substructure I (ring a), substructure II (ring b), and substructure III (aliphatic chain c). Typically, substructures I and II in each compound were linked together via substructure III. A closer look at the structures of compounds **1**–**6** revealed that these six acylphenols were structurally related to one another.

Compounds **1**–**4**

Substructures I and III were common in all four acylphenols and were, respectively, identified as a 2-acylresorcinol moiety and an *n*-octyl chain. Variations were, however, observed in substructure II. Unlike compound **1,** whose substructure II was a monosubstituted aromatic ring, those of compounds **2**–**4** were either 1,4-disubstituted (**2**) or 1,3,4-trisubstituted (**3** and **4**) aromatic rings. Furthermore, positions C-12 and C-13 in substructure II in compounds **2**–**4** were each occupied by oxygenated functional groups such as hydroxyl (**2** and **3**) and methylenedioxy (**4**) groups in contrast to compound **1,** whose substructure II does not have any oxygenated functional groups bonded to it. Based on this review, these acylphenols are ubiquitous in the genus *Myristica* in particular compounds **2** and **3** (Table 1, Figure 1).

Compound **5**

Until present, compound **5** has only been isolated and characterized from the fruits of *M. cinnamomea* (Table 1, Figure 1), thus making it a potential chemotaxonomic marker for this particular Malaysian *Myristica* species. The structure of compound **5** closely resembled that of compound **2** with the only difference being in its substructure I. In compound **5**, its 2-acylresorcinol moiety has a hydroxyl group at position C-19 in addition to the hydroxyl groups at positions C-17 and C-21, thus making its substructure I a 1, 2, 3, 5-tetrasubstituted symmetrical aromatic ring instead of a 1, 2, 3-trisubstituted symmetrical aromatic ring, as in compound **2**.

Compound **6**

Though compound **6** is a known acylphenol, it was identified in the bark of *M. fatua* Houtt. var. *magnifica* for the first time in 2018 (Table 1, Figure 1). Its structure was almost identical to that of compound **1**, with the only difference being in its substructure I. Unlike in compound **1**, whose position C-21 bore a hydroxyl group, the corresponding position in compound **6** was occupied by a methoxyl group instead.

Type 2 Acylphenols

Compounds **7** and **8**, classified as Type 2 acylphenols, were isolated and characterized from the fruit rinds of *M. beddomei* subsp. *sphaerocarpa* and *M. malabarica* (Table 1, Figure 1). Similar to Type 1 acylphenols, the basic skeleton of Type 2 acylphenols was also constructed from three substructures: substructure I (ring a) and substructure II (ring b), which were linked together via substructure III (aliphatic chain c). It is interesting to note that there was a striking difference between the type of ring which constituted substructure I in Types 1 and 2 acylphenols. It was evident that compounds **7** and **8** were structurally related to one another. Substructure I, an 1-acyl-4-hydroxycyclohexane-2,6-dione ring and substructure III, an *n*-octyl chain were common in both the acylphenols. Substructure II, however, differed between them. While substructure II was a 1,4-disubstituted aromatic ring with a hydroxyl group at position C-13 in compound **7**, the corresponding substructure in compound **8** was a 1,3,4-trisubstituted aromatic ring with positions C-12 and C-13 being occupied by hydroxyl groups.

Type 3 Acylphenols

Compounds **9** and **10,** classified as Type 3 acylphenols, were obtained from the bark and the various parts of the fruits of *M. beddomei* subsp. *Sphaerocarpa*, *M. fatua*, *M. fatua* var. *magnifica*, and *M. malabarica* (Table 1, Figure 1). Type 3 acylphenols were significantly different compared to Types 1 and 2 acylphenols. The former was only constructed from two substructures instead of three: substructure I (ring a) and substructure II (aliphatic chain b). Substructures I and II in compound **9** were, respectively, identified as a 2-acylresorcinol moiety and an *n*-tridecyl chain. Compound **10** was structurally similar to compound **9**. However, position C-20 of its 2-acylresorcinol moiety bore a methoxyl group in place of a hydroxyl group.

Type 4 Acylphenols

Compound **11** was isolated and characterized from the bark of *M. cinnamomea* (Table 1, Figure 1). It was an acylphenol with a novel skeleton comprising four substructures: substructure I (ring a), substructure II (ring b), substructure III (aliphatic chain c), and substructure IV (ring d). Substructures I and IV were, respectively, identified as a 2-acylresorcinol moiety and a dihydropyran-4-one moiety, and both of these substructures were fused together. Substructure II, on the other hand, was a 1,4-disubstituted aromatic ring with a hydroxyl group bonded to position C-13. These three substructures were linked together via substructure III, a *n*-hexyl chain.

Type 5 Acylphenols

This novel group of compounds (**12**–**19**) was recently isolated and characterized from the seeds of *M. fragrans* (Table 1, Figure 1). These eight compounds could be further divided into three sub-groups based on the classes of compounds, which occupied positions C-12, C-13, and C-18 of the acylphenol unit.

Group 1

Compounds **12** and **13** were constructed from an acylphenol (compound **3**), a 8-O-4′ type neolignan (myrisisolignan), and an oxygenated monoterpenoid (terpinen-4-ol). 

In compound **12**, the neolignan unit was bonded to position C-18 of the acylphenol unit via a carbon–carbon linkage. The monoterpenoid unit, on the other hand, was bonded via an ether linkage to position C-12 of the acylphenol unit. 

The structure of compound **13** was almost identical to compound **12**. The monoterpenoid unit in compound **13**, however, occupied position C-13 of the acylphenol unit, unlike in compound **12**.

Group 2

The structure of compound **14** was significantly different from compound **13**. Though the monoterpenoid unit (terpinen-4-ol) in compound **14** was bonded to position C-13 of the acylphenol unit (compound **3**), similar to compound **13**, position C-18 of the acylphenol unit in compound **14** was, however, occupied by a phenylpropanoid unit instead of a 8-O-4′ type neolignan unit.

Group 3

The structures of compounds **15**–**19** differed notably from those of compounds **12**–**14**. The former were constructed only from an oxygenated monoterpenoid (either terpinen-4-ol or alpha-terpineol), which was bonded to either position C-12 or C-13 of an acylphenol (either compound **2** or **3**) via an ether linkage.

##### Dimeric Acylpenols

The dimeric acylphenols (**20**–**25**) were isolated and characterized from the bark and fruits of *M. cinnamomea* and *M. maxima* (Table 1, Figure 2). They could be further divided into two types: Type 1 (**20**–**23**) and Type 2 (**24** and **25**).

Type 1 Dimeric Acylphenols

Compounds **20**–**22** were structurally related to each other and were derived from the following acylphenols; compounds **1**–**3**. While compound **20** was identified to be a dimer of compound **3**, compound **22** was characterized as a dimer of compounds **2** and **3.** As for compound **21**, though it was established as a new dimeric acylphenol, which was obtained from the bark of *M. cinnamomea*, it was constructed from the two known acylphenols: compounds **1** and **3**. 

The bark of *M. maxima* also yielded a new dimeric acylphenol, compound **23**, which was a positional isomer of compound **20**. Compounds **20** and **23** only differed from one another in the position of the hydroxyl groups in their respective ring b. Unlike compound **20**, whose ring b bore ortho-dihydroxyl groups with the hydroxyl groups being at positions C-12 and C-13, the corresponding substructure in compound **23** bore meta-dihydroxyl groups, in which the hydroxyl groups were bonded to positions C-12 and C-14 instead. It is interesting to note that compound **23** featured a new acylphenol as one of its monomers that is yet to be identified in the plant kingdom, while its other monomer was compound **3**.

Compounds **20**–**23** were all non-symmetrical in nature. Their monomers (acylphenols) were linked together via a carbon–carbon linkage between the aromatic rings in each monomer.

Type 2 Dimeric Acylphenols

Compounds **24** and **25** were structurally related to each other and were constructed from the following acylphenols: compounds **2** and **3**. Compound **24** was derived from compound **3**, while compound **25** was derived from compounds **2** and **3**.

Compounds **24** and **25** were non-symmetrical in nature. The monomers (acylphenols), which constituted each dimeric acylphenol, were linked together via a carbon–carbon linkage between an aromatic ring of one monomer and the *n*-octyl chain of the other monomer.

### 4.2. Pharmacological Activities of Myristica *spp.*

The acylphenols and dimeric acylphenols from this genus have been shown to have a broad spectrum of pharmacological activities, which are summarized in Table 3, Table 4, Table 5, Table 6, Table 7, Table 8, Table 9, Table 10, Table 11, Table 12 and Table 13 and will be discussed in the following sections. 

#### 4.2.1. Antioxidant Activity

Othman et al. (2016) demonstrated compounds **3**, **20**, **23**, **24**, and **25** to be 4 to 9 folds more potent free radical scavengers compared to ascorbic acid (Table 3). They subsequently concluded that the number of hydroxyl groups present in ring b in the acylphenols and ring b’ in the dimeric acylphenols and the position of the hydroxyl groups in ring b in the dimeric acylphenols could have contributed to the differences in their scavenging potentials (Figure 1 and Figure 2). Othman et al. further postulated that dimerization could have increased the scavenging capacity of compound **20** in comparison to its monomer compound **3**. Compound **20** possesses double the number of hydroxyl groups as compared to compound **3**. Therefore, with a larger conjugated system, the electron withdrawing effects in compound **20** could have led to the easy oxidation of the hydroxyl groups. 

**Table 3:** The antioxidant activities of acylphenols and dimeric acylphenols from the genus *Myristica*.

*Myristica* spp.	Part of the Plant Investigated	Acylphenols/Dimeric Acylphenols	Main Findings	Reference
*M. fragrans* Houtt.	Aril	Malabaricone C (**3**)	**Antioxidant activity: In vitro DPPH free radical scavenging activity** **3**: IC_50_ = 6.56 ± 0.02 μg/mL Ascorbic acid (positive control): IC_50_ = 5.76 ± 0.01 μg/mL	[44]
*M. fragrans* Houtt.	Seeds	Malabaricone B (**2**) Malabaricone C (**3**)	**Antioxidant activity: In vitro DPPH free radical scavenging activity** **2**: IC_50_ = >200 μg/mL **3**: IC_50_ = 8.35 ± 2.20 μg/mL BHT (positive control): IC_50_ = 34.28 ± 1.40 μg/mL **Antioxidant activity: In vitro ABTS radical scavenging activity** **2**: IC_50_ = 7.05 ± 0.72 μg/mL **3**: IC_50_ = 5.36 ± 0.19 μg/mL BHT (positive control): IC_50_ = 10.67 ± 0.41 μg/mL **Antioxidant activity: In vitro Hydroxyl radical scavenging activity** **2**: IC_50_ = 95.22 ± 4.20 μg/mL **3**: IC_50_ = 72.81 ± 2.58 μg/mL BHT (positive control): IC_50_ = 69.96 ± 4.66 μg/mL **Antioxidant activity: In vitro Superoxide radical scavenging activity** **2** and **3**: No significant effect. BHT (positive control): No available data.	[10]
*M. maxima* warb.	Bark	Malabaricone A (**1**) Malabaricone B (**2**) Malabaricone C (**3**) Giganteone A (**20**) Giganteone E (**22**) Maingayone A (**24**) Maingayone B (**25**)	**Antioxidant activity: In vitro free radical scavenging activity** **1**: IC_50_ = 522.76 ± 1.45 μM **2**: IC_50_ = 340.93 ± 1.19 μM **3**: IC_50_ = 5.28 ± 0.05 μM **20**: IC_50_ = 3.17 ± 0.07 μM **22**: IC_50_ = 2.92 ± 0.10 μM **24**: IC_50_ = 2.90 ± 0.01 μM **25**: IC_50_ = 6.08 ± 0.20 μM Ascorbic acid (positive control): IC_50_ = 26.25 ± 0.34 μM.	[56]

Li et al. (2020) revealed that compound **3** was a stronger DPPH, ABTS, and hydroxyl radical scavenger compared to compound **2** (Table 3). Compound **3** was also found to be, respectively, 4.1 folds and 2.0 folds more effective in scavenging the DPPH and ABTS radicals compared to BHT. As for its ability to scavenge the hydroxyl radical, compound **3** was found to be comparable to that of BHT (Table 3). The potency of compound **3** compared to that of compound **2** may have been attributed to the presence of the two hydroxyl groups in its ring b, unlike the latter, which only bore a single hydroxyl group in its corresponding substructure (Figure 1).

Sathya et al. (2020) found compound **3** to have a comparable free radical scavenging potential as ascorbic acid (Table 3). 

#### 4.2.2. Anti-Inflammatory Activity

Basak et al. (2020) recently examined the effects of compound **3** on the nonsteroidal anti-inflammatory drug (NSAID)-induced gastrointestinal damage (Table 4). The dose dependent treatment of compound **3** improved the histopathological appearance of indomethacin-induced mucosal lesions and gastric inflammation in male Swiss albino mice. At a dosage of 10 mg/kg, compound **3** decreased gastric inflammation following the exposure to indomethacin more effectively compared to omeprazole (3 mg/kg), after three days of treatment (Table 4). By decreasing oxidative and nitrative stress, treatment with compound **3** prevented NSAID-induced mitochondrial dysfunction and cell death; nuclear factor j-light-chain enhancer of activated B cell induction; the release of proinflammatory cytokines and neutrophil infiltration; and disruptions in the vascular endothelial growth factor/endostatin balance that contributed to mucosal auto healing. Importantly, compound **3** failed to impact the therapeutic anti-inflammatory properties of multiple NSAIDs in a model of acute inflammation. In all assays tested, compound **3** proved as or more efficacious than the current first-line therapy for NSAID-dependent GI complications, the proton pump inhibitor omeprazole.

**Table 4:** The anti-inflammatory activities of acylphenols from the genus *Myristica*.

*Myristica* spp.	Part of the Plant Investigated	Acylphenols	Main Findings	Reference
*M. fragrans* Houtt.	Fruits	Malabaricone C (**3**)	**Anti-Inflammatory activity: In vitro Inhibition of the 5-lipoxygenase enzyme** **3**: IC_50_ = 0.2 μM **In vivo imiquimod-induced psoriasis-like skin lesion** Topical application with 2 mM of compound **3** significantly ameliorated hyperplasia and inflammatory cell infiltration. Compound **3** markedly decreased the level of LTB4 but did not significantly increase the level of other pro-inflammatory lipid mediators.	[45]
*M. malabarica* Lam.	Rind	Malabaricone C (**3**)	**Anti-inflammatory activity: Histological inflammatory scores against indomethacin-induced stomach ulceration in mice (*n* = 10)** Day 1 Control: 1.9 Indomethacin + **3** (2 mg/kg): 1.7 Indomethacin + **3** (5 mg/kg): 1.5 Indomethacin + **3** (10 mg/kg): 1.1 Indomethacin + **3** (15 mg/kg): 0.9 Indomethacin + **3** (20 mg/kg): 0.8 Day 3 Control: 2.7 Indomethacin + **3** (2 mg/kg): 1.7 Indomethacin + **3** (5 mg/kg): 0.9 Indomethacin + **3** (10 mg/kg): 1.0 Indomethacin + **3** (15 mg/kg): 0.7 Indomethacin + **3** (20 mg/kg): 0.6 Day 5 Control 1.3 Indomethacin + **3** (2 mg/kg): 1.1 Indomethacin + **3** (5 mg/kg): 0.8 Indomethacin + **3** (10 mg/kg): <0.5 Indomethacin + **3** (15 mg/kg): <0.5 Indomethacin + **3** (20 mg/kg): <0.5 Day 7 Control: 0.9 Indomethacin + **3** (2 mg/kg): 0.5 Indomethacin + **3** (5 mg/kg): <0.5 Indomethacin + **3** (10 mg/kg): <0.5 Indomethacin + **3** (15 mg/kg): <0.5 Indomethacin + **3** (20 mg/kg): <0.5 Compound **3** decreases gastric inflammation following indomethacin exposure with improved efficacy over omeprazole. Compound **3** protects against oxidative stress induced by indomethacin in the stomach. Compound **3** ameliorates indomethacin-induced cell death and inflammation by decreasing oxidative and nitrative stress. Indomethacin-induced, NF-κβ-mediated MMP-9 activation, and inflammatory cytokine production are decreased by compound **3**. Indomethacin-dependent increases in endostatin and decreases in VEGF levels are reversed by compound **3**. Compound **3** improves stomach ulceration following indomethacin exposure without impacting the anti-inflammatory properties of the drug.	[53]

Recently, Tsukayama et al. (2022) demonstrated that compound **3** exhibited potent (IC_50_ = 0.2 μM) competitive inhibition of the 5-LOX enzyme (Table 4). A concentration between 0–0.5 μM of compound **3** was also found to dose dependently reduce the production of LTB_4_ in the RBL-2H3 cells without having any effect on the cell viability. LTB_4_ is derived from arachidonic acid via the LOX pathway and is an important proinflammatory mediator. Studies have demonstrated that elevated levels of LTB_4_ are linked to the pathogenesis of several inflammatory diseases [58]. Tsukayama et al., subsequently, investigated the effects of compound **3** in ameliorating the imiquimod-induced skin inflammation in BALB/c mice (Table 4). Their study revealed that the topical application of compound **3** (2 mM) significantly ameliorated hyperplasia and inflammatory cell infiltration and suppressed the expression of the psoriasis-associated genes S100a9, Krt1, Il17a, and Il22. Lipid metabolome analysis of these psoriasis-like skin lesions showed that compound **3** markedly decreased the level of LTB_4_ but did not significantly increase the levels of other pro-inflammatory lipid mediators.

#### 4.2.3. Antiproliferative and Cytotoxic Activities

Tyagi et al. (2014a) reported the cytotoxicity of compound **3**, isolated from the methanol extract of the fruit rinds of *M. malabarica*, using the photometric enzyme immunoassay to quantify the formation of cytoplasmic histones associated DNA fragments (mono and oligosomes) after the apoptotic cell death. Compound **3** was found to dose dependently inhibit the growth of the MCF-7 cells more effectively compared to curcumin after 48 h of incubation (Table 5). The pro-apoptotic mechanism of compound **3** with the MCF-7 cells involved the deregulation of multiple targets associated with the mitochondria, leading to the activation of different enzymatic cascades. 

**Table 5:** The antiproliferative and cytotoxic activities of acylphenols and dimeric acylphenols from the genus *Myristica*.

*Myristica* spp.	Part of the Plant Investigated	Acylphenols/Dimeric Acylphenols	Main Findings	Reference
*M. beddomei* subsp. *sphaerocarpa* W.J. de Wilde	Rind/Seeds/Bark	Malabaricone A (**1**) Malabaricone B (**2**) Malabaricone C (**3**) Malabaricone D (**4**) Promalabaricone B (**7**)	**Cytotoxic activity: In vitro cytotoxic activity against human breast adenocarcinoma cancer cell lines (MCF-7 and MDA-MB-231) and normal cell line (WI 38)** MCF-7 **1**: IC_50_ = 15.4 μg/mL **2**: IC_50_ = 22.92 μg/mL **3**: IC_50_ = 36.25 μg/mL **4**: IC_50_ = 20.58 μg/mL **7**: IC_50_ = 74.41 μg/mL Doxorubicin (positive control): IC_50_ = >100 μg/mL MD-AMB-231 **1**: IC_50_ = 28.58 μg/mL **2**: IC_50_ = 14.67 μg/mL **3**: IC_50_ = 31.25 μg/mL **4**: IC_50_ = 32.87 μg/mL **7**: IC_50_ = 86.12 μg/mL Doxorubicin (positive control): IC_50_ = 31.90 μg/mL WI 38 **1**: IC_50_ = 25.37 μg/mL **2**: IC_50_ = 35.13 μg/mL **3**: IC_50_ = >100 μg/mL **4**: IC_50_ = 31.80 μg/mL **7**: IC_50_ = >100 μg/mL Doxorubicin (positive control): IC_50_ = No data available	[35]
*M. fatua* Houtt.	Bark	Malabaricone B (**2**) Malabaricone C (**3**)	**Cytotoxic Activity: In vitro cytotoxic activity against human breast adenocarcinoma cancer cell line (MCF-7)** **2**: IC_50_ = 0.71 μg/mL **3**: IC_50_ = 2.38 μg/mL	[39]
*M. fragrans* Houtt.	Seeds	Malabaricone C (**3**)	**Cytotoxic activity: In vitro cytotoxic activity against human gastric cancer cell lines (NCIN87 and MGC803)** NCIN87 **2**: IC_50_ = 19.80 ± 1.70 μg/mL **3**: IC_50_ = 42.62 ± 3.10 μg/mL Vinorelbine (positive control): 20.06 ± 1.91 μg/mL MGC803 **2**: IC_50_ = 19.60 ± 2.21 μg/mL **3**: IC_50_ = 22.94 ± 1.33 μg/mL Vinorelbine (positive control): 18.65 ± 2.23 μg/mL	[14]
*M. fragrans* Houtt.	Seeds	Malabaricone A (**1**) Myrifratin A (**12**) Myrifratin B (**13**) Myrifratin C (**14**) Myrifratin D (**15**) Myrifratin E (**16**) Myrifratin F (**17**) Myrifratin G (**18**) (−)-1-(2,6-dihydrox- yphenyl)-9-[4-hydroxy-3-(p-menth-1-en-8-oxy)-phenyl]-1-nonanone (**19**)	**Autophagy modulating activities** Compounds **1**, **15**−**17** and **19** accumulated GFP-LC3 puncta in HEK293 cells. Compounds **15** and **16** induced GFP-LC3 puncta and upregulated the protein expressions of autophagy markers (LC3-II and p62).	[47]
*M. malabarica* Lam.	Rind	Malabaricone C (**3**)	**Cytotoxic activity: In vitro cytotoxic activity against human breast cancer cell line (MCF-7)** After 48 h of treatment: **3**: IC_50_ = 7.0 ± 1.8 μM Curcumin (positive control): IC_50_ = 19.7 ± 2.5 μM Compound **3** induces ΔΨm loss to release the mitochondrial nucleases in MCF-7 cells. Compound **3** increases intracellular Ca^2+^ levels and activates calpain in MCF-7 cells. Compound **3** induces LMP to release cathepsin B and activate Bid in MCF-7 cells. Compound **3** arrests the S and G2-M phases in MCF-7 cells.	[48]
*M. malabarica* Lam.	Rind	Malabaricone A (**1**) Malabaricone B (**2**) Malabaricone C (**3**) Malabaricone D (**4**)	**Cytotoxic activity: In vitro cytotoxic activity against lung carcinoma cell lines (A549, NCI-H460, NCI-H23 and NCI-H522)** A549 after 48 h of treatment: **1**: IC_50_ = 19.2 ± 4.2 μM **2**: IC_50_ = 8.4 ± 2.5 μM **3**: IC_50_ = 7.0 ± 1.8 μM **4**: IC_50_ = 20.3 ± 5.1 μM Curcumin (positive control): IC_50_ = 41.7 ± 6.2 μM NCI-H460 after 48 h of treatment: **3**: IC_50_ = 7.7 ± 2.1 μM Curcumin (positive control): IC_50_ = 27.3 ± 4.2 μM NCI-H23 after 48 h of treatment: **3**: IC_50_ = 9.9 ± 2.7 μM Curcumin (positive control): IC_50_ = 22.8 ± 4.0 μM NCI-H522 after 48 h of treatment: **3**: IC_50_ = 12.4 ± 3.4 μM Curcumin (positive control): IC_50_ = 26.2 ± 3.6 μM Compound **3** perturbs mitochondrial function through BAX/BCL-2 imbalance. Compound **3** binds to DNA and induces DSBs. Compound **3** induces ATM/ATR-mediated DNA damage response and *p38 MAPK* activation.	[13]
*M. malabarica* Lam.	Rind	Malabaricone A (**1**)	**Cytotoxic activity: In vitro cytotoxic activity against leukemic cancer cell lines (MOLT3, K562 and HL-60) and solid tumor cell lines (MCF7, A549 and HepG2)** MOLT3 after 48 h of treatment: **1**: IC_50_ = 17.20 ± 2.22 μg/mL K562 after 48 h of treatment: **1**: IC_50_ = 18.10 ± 0.95 μg/mL HL-60 after 48 h of treatment: **1**: IC_50_ = 12.70 ± 0.46 μg/mL MCF7 after 48 h of treatment: **1**: IC_50_ = 32.95 ± 1.63 μg/mL A549 after 48 h of treatment: **1**: IC_50_ = 55.26 ± 5.90 μg/mL HepG2 after 48 h of treatment: **1**: IC_50_ = 28.10 ± 0.58 μg/mL Compound **1** mediated cytotoxicity in leukemic cell lines via generation of a redox imbalance. Compound-**1**-induced mitochondrial apoptotic events were higher in MOLT3 than in MCF7 and A549. Compound **1** down regulated Nrf2 signaling pathway.	[49]
*M. malabarica* Lam.	Rind	Malabaricone A (**1**)	**Cytotoxic activity: In vitro cytotoxic activity against T-lymphoblastic leukemic cell line, CCRF CEM and its multidrug resistance (MDR) counterpart, CEM/ADR5000)** CCRF CEM after 48 h of treatment: **1**: IC_50_ = 9.72 ± 1.08 μg/mL CEM/ADR5000 after 48 h of treatment: **1**: IC_50_ = 5.40 ± 1.41 μg/mL Compound-**1**-mediated cytotoxicity was via generation of ROS. Compound **1** induced depletion of the antioxidant component. Compound **1** caused comparable caspase-3 activity.	[50]
*M. malabarica* Lam.	Rind	Malabaricone A (**1**)	**Cytotoxic activity: In vitro cytotoxic activity against hematopoietic U937 and MOLT3 cell lines** U937 after 48 h of treatment: **1**: IC_50_ = 15.38 ± 1.91 μg/mL MOLT3 after 48 h of treatment: **1**: IC_50_ = 17.42 ± 0.47 μg/mL Compound **1** caused a minimal increase in the phosphorylation of PTEN, and a substantial time-dependent dephosphorylation of AKT and mTOR. Compound-**1**-induced generation of ROS was mediated via activation of the MAPK (p38 and JNK) pathway, along with inhibition of the PI3K/AKT pathway.	[51]
*M. malabarica* Lam.	Rind	Malabaricone B (**2**)	**Cytotoxic activity: In vitro cytotoxic activity against human cancer cell lines** A549, human lung cancer **2**: IC_50_ = 8.1 ± 1.0 μM Curcumin (positive control): IC_50_ = 26.7 ± 3.1 μM A375, malignant melanoma **2**: IC_50_ = 26.7 ± 2.9 μM Jurkat, T cell leukemia **2**: IC_50_ = 27.4 ± 3.1 μM A431, epidermoid carcinoma **2**: IC_50_ = 9.5 ± 3.2 μM NCI-H23, lung adenocarcinoma **2**: IC_50_ = 9.6 ± 1.2 μM K562, chronic myelogenous leukemia **2**: IC_50_ = 47.0 ± 3.9 μM U937, leukemic monocyte lymphoma **2**: IC_50_ = 27.5 ± 1.4 μM MCF-7, breast carcinoma **2**: IC_50_ = 9.3 ± 2.1 μM Compound **2** activates caspases-9 and 3, but not caspase-8. Compound **2** induces mitochondrial ΔΨm and triggers intracellular ROS generation to induce apoptosis. Compound-**2**-induced cytotoxicity is regulated by BAX/BCL-2. Compound **2** reduces lung tumor (xenograft) burden in mice.	[59]
*M. malabarica* Lam.	Rind	Malabaricone C (**3**)	**Cytotoxic activity** **3**: Increasing time (1.5 h, 3 h, and 6 h) and concentration (0 μM, 4 μM, 6 μM, and 8 μM) dependent on ROS generation. **3** (6 μM): Pretreatment of the cells with intracellular ROS scavengers such as NAC, PEG-SOD, PEGCAT, Na-pyurvate, tocopherol, Trolox^®^, ascorbate, and cell permeable SOD-mimetic (Mn-TBAP) attenuated the ROS level by 81%, ~34%, 25%, 20%, 39%, 46%, 53%, and 50%. **3** (6 and 8 μM): Decreased free GSH content of the cells by ~45% and 53%. NEM (0.5 mM): Decreased free GSH by 88.7%. NAC augments compound-**3**-induced DNA damage and oxidative stress. Thiol antioxidants modulate pro-survival signaling in compound-**3**-induced death process. NAC enhances S-glutathionylation of p65 and p53 proteins in response to treatment with compound **3**. **In vivo tumor growth in mice** Compound **3** reduces the growth of lung tumor in a xenograft model and the growth of a highly metastatic melanoma tumor in a syngeneic mouse model. Compound **3** with (NAC) combination may effectively manage secondary lung tumors arising from melanoma metastasis.	[60]
*M. malabarica* Lam.	Rind	Malabaricone A (**1**) Malabaricone B (**2**) Malabaricone C (**3**) Malabaricone D (**4**) Promalabaricone B (**7**) Promalabaricone C (**8**) 1-(2,6-dihydroxyphenyl)tetradecan-1-one (**9**)	**Anti-proliferative activity: In vitro cytotoxic activity against human ovarian cancer cell line (A2780)** **1**: IC_50_ = 2.5 ± 0.2 μM **2**: IC_50_ = 5.5 ± 0.5 μM **3**: IC_50_ = 2.3 ± 0.2 μM **4**: IC_50_ = 8.1 ± 0.5 μM **7**: IC_50_ = 2.2 ± 0.2 μM **8**: IC_50_ = 2.0 ± 0.5 μM **9**: IC_50_ = 2.0 ± 0.2 μM Paclitaxel (positive control): IC_50_ = 0.037 μM	[55]
*M. maxima* Warb.	Bark	Malabaricone A (**1**) Malabaricone B (**2**) Malabaricone C (**3**) Giganteone A (**20**) Giganteone E (**23**) Maingayone A (**24**) Maingayone B (**25**)	**Cytotoxic activity: In vitro cytotoxic activity against human prostate cancer cell line (PC3).** After 24 h of treatment: **1**: IC_50_ = 26.0 ± 3.3 μM **2**: IC_50_ = 73.4 ± 3.9 μM **3**: IC_50_ = 143.1 ± 2.8 μM **20**: IC_50_ = 17.5 ± 1.7 μM **23**: IC_50_ = >200 μM **24**: IC_50_ = 31.6 ± 5.3 μM **25**: IC_50_ = 124.7 ± 5.2 μM Doxorubicin (positive control): IC_50_ = 9.7 ± 2.2 μM After 48 h of treatment: **1**: IC_50_ = 9.2 ± 2.4 μM **2**: IC_50_ = 31.8 ± 3.2 μM **3**: IC_50_ = 50.5 ± 2.1 μM **20**: IC_50_ = 6.3 ± 1.2 μM **23**: IC_50_ = 151.1 ± 4.5 μM **24**: IC_50_ = 13.4 ± 4.6 μM **25**: IC_50_ = 80.6 ± 8.0 μM Doxorubicin (positive control): IC_50_ = 2.3 ± 1.2 μM	[56]

Later, in the same year, Tyagi et al. (2014b) found compounds **1**–**4** to have significant cytotoxic activity against several types of lung carcinoma cell lines, among which include A549, NCI-H460, NCI-H23, and NCI-H522 (Table 5). Compounds **1**–**4** dose dependently induced cell death more effectively in the A549 cells compared to curcumin, with IC_50_ values of 19.2 ± 4.2 μM (**1**), 8.4 ± 2.5 μM (**2**), 7.0 ± 1.8 μM (**3**), 20.3 ± 5.1 μM (**4**), and 41.7 ± 6.2 μM (curcumin) after 48 h of incubation. Interestingly, compound **3** induced maximum cell death even at 24 h with no further increase with time. Compound **3** also reduced the viability of the NCI-H460, NCI-H23, and NCI-H522 cell lines more effectively than curcumin (Table 5). Compound **3** activated the ATM-CHK1-p38 MAPK cascade to cause mitochondrial cell death in the lung carcinoma cells. 

Wu et al. (2014) investigated the cytotoxic effects of acylphenols on the human gastric cancer cell lines (NCIN87 and MGC803) using the MTT assay (Table 5). Compounds **2** and **3** were both able to inhibit the cytotoxic activities of the NCIN87 and MGC803 cell lines. However, compound **2** was more cytotoxic compared to compound **3,** and it exhibited a cytotoxic activity almost similar to that of vinorelbine. The molecular mechanism of the cytotoxicity of these acylphenols on these human gastric cancer cell lines, however, is unclear.

Manna et al. (2015a) screened the cytotoxic potential of compound **1** against leukemic cell lines (MOLT3, K562 and HL-60) and compared its activity against solid tumor cell lines (MCF7, A549 and HepG2) (Table 5). Compound **1** demonstrated higher cytotoxicity against all three leukemic cell lines than in the solid tumor cell lines. With regard to the leukemic cell lines, the IC_50_ values of compound **1** ranged from 12.70 ± 0.46 μg/mL to 18.10 ± 0.95 μg/mL, whereas for the solid tumor cell lines, the IC_50_ values were higher and ranged from 28.10 ± 0.58 μg/mL to 55.26 ± 5.90 μg/mL (Table 5). The higher degree of cytotoxicity against MOLT3 was due to a higher induction of redox imbalance, evident from an increased generation of ROS and a concomitant depletion of thiols. This was confirmed by pre-incubation with NAC and BSO, wherein NAC decreased compound-**1**-induced cytotoxicity by 2.04-folds, while BSO enhanced the cytotoxicity and decreased the IC_50_ value by 5.60-folds. 

In the same year, Manna et al. (2015b) also evaluated the cytotoxic potential of compound **1** against the T-lymphoblastic leukemic cell line, CCRF CEM, and its multidrug resistance (MDR) counterpart CEM/ADR5000 (Table 5). The cytotoxicity of compound **1** was 1.8-folds higher against the CEM/ADR5000 cell line than against the CCRF CEM cell line, suggesting that compound **1** demonstrated “collateral sensitivity”. This cytotoxicity of compound **1** was attributed to an enhanced generation of oxidative stress, as the IC_50_ value increased following the addition of an antioxidant N-acetyl cysteine (NAC). Furthermore, compound **1** depleted glutathione and inhibited the activity of glutathione peroxidase, which also contributed to the generation of a redox imbalance. 

The following year, Manna et al. (2016) reported on the cytotoxic activity of compound **1** against two hematopoietic cell lines U937 and MOLT3 (Table 5). Manna and his coworkers demonstrated that the IC_50_ values of compound **1** against both cell lines were 15.38 ± 1.91 μg/mL and 17.42 ± 0.47 μg/mL, respectively (Table 5). Compound **1** enhanced the phosphorylation of the components of the pro-apoptotic pathway, namely ASK1, p38, and JNK while decreasing the phosphorylation of AKT and mTOR. The cytotoxicity of compound **1** was attenuated by the inhibitors of p38 and JNK, whereas it was enhanced in the presence of a PI3K/AKT inhibitor.

Othman et al. (2016) examined the cytotoxic potential of the acylphenols (**1**–**3**) and dimeric acylphenols (**20**, **23**, **24** and **25**) isolated from the bark of *M. maxima* against human prostate cancer cell lines, PC3 (Table 5). The results from their MTT assay revealed that only compounds **1**, **20,** and **24** were active against the PC3 cell lines after 48 h of treatment (Table 5). Othman and his coworkers concluded that the increase in the number of hydroxyl groups in ring b in the acylphenols could have decreased their cytotoxic potential (Figure 1). Othman et al. deduced that the potency of compound **20** compared to compound **23** could have been attributed to the presence of the ortho-dihydroxyl groups in the ring b of the former in contrast to the latter, whose ring b bore meta-dihydroxyl groups instead (Figure 2). Othman et al. further postulated that the stronger cytotoxicity of compound **24** compared to that of compound **25** may have resulted from the presence of the two hydroxyl groups in its ring b’ unlike the latter which only bore a single hydroxyl group in its ring b′ (Figure 2).

Megawati et al. (2017) assessed the cytotoxic activity of compounds **2** and **3** isolated from *M. fatua* against the breast carcinoma cancer cell line MCF-7, using the Alamar blue assay. Both compounds exhibited strong to potent cytotoxicity activity with IC_50_ values of 0.71 μg/mL and 2.38 μg/mL, respectively (Table 5). 

Tyagi et al. (2018) evaluated the cytotoxic activity of compound **2** isolated from *M. malabarica* against a panel of eight human cancer cell lines: lung carcinoma (A549), malignant melanoma (A375), T cell leukemia (Jurkat), epidermoid carcinoma (A431), lung adenocarcinoma (NCI-H23), chronic myelogenous leukemia (K562), leukemic monocyte lymphoma (U937), breast carcinoma (MCF-7), and three normal cell lines: intestinal (INT 407), lung fibroblast (WI-38), and embryonic kidney (HEK293) (Table 5). Compound **2** showed selective toxicity towards the A549, A375, and Jurkat cells without showing toxicity towards the INT407, HEK293, and WI-38 cells. Among the tested cell lines, compound **2** was found to exhibit the strongest cytotoxic activity against the A549 cell line, following which it was found to be 3.2 folds more potent than curcumin (Table 5). Compound-**2**-induced apoptosis was mediated by an increase in the intracellular reactive oxygen species (ROS), as a result of the cell-permeable antioxidants, N-acetylcysteine (NAC), and PEG-SOD, which strongly inhibited its cytotoxicity towards the A549 cells. Compound **2** increased the BAX level while simultaneously decreasing the BCL-2 and BCL-XL levels in the A549 cells, in turn, triggering the mitochondrial apoptotic pathway as revealed from the release of cytochrome c and the activation of caspase-9 and caspase-3. Pre-treatment of the A549 cells with caspase-9, caspase-3, and pan-caspase inhibitors made them more resistant to the treatment with compound **2**. This effect of compound **2** was strongly associated with the concomitant decrease in anti-apoptotic (IAP1, IAP2, and survivin), angiogenic (growth factors), and cancer invasiveness (matrix metalloproteinase-9, COX-2) modulating proteins. Compound-**2**-induced cytotoxicity was unaffected by the shRNA-mediated depletion of p53 in the A549 cells.

Subsequently, Tyagi et al. (2020) demonstrated that compound **3** with N-acetyl cysteine (NAC) was a promising therapeutic regimen for lung cancer treatment in vitro and in vivo (Table 5). Compound **3** reduces lung tumor growth in a xenograft model and highly metastatic melanoma tumor growth in a syngeneic mouse model. Combination of compound **3** with (NAC) may effectively manage secondary lung tumors arising from melanoma metastasis. 

A year later, Neethu et al. (2021) investigated the cytotoxic effects of acylphenols (**1**–**4** and **7**) against the human breast adenocarcinoma cell lines MCF-7 and MDAMB-231 along with the normal cell line WI 38, using the thiazolyl blue tetrazolium bromide (MTT) assay (Table 5). Compounds **1**–**4** showed promising cytotoxic activity against the MCF-7 and MD-AMB -231 cell lines but showed moderate toxicity against the normal cells. Their IC_50_ values were comparable to or better than the standard Doxorubicin. 

Recently, Bauri et al. (2022) conducted an in vitro assay to determine the cytotoxic potential of the acylphenols (compounds **1**–**4** and **7**–**9**) from *M. malabarica* against the human ovarian cancer cell lines A2780. All six compounds exhibited moderate anti-proliferative activity as compared to paclitaxel. It is noteworthy to mention that compounds **1**, **3**, **7**, **8,** and **9** possessed almost similar cytotoxicity (Table 5).

Shen et al. (2022) examined the autophagy modulating activities of compounds **1** and **12**–**19**, which they had recently identified in the seeds of *M. fragrans*. The assay was conducted using the HEK293-GFP-LC3 cell lines. After treatment of the HEK293-GFP-LC3 cells with 40 μM of compounds **1**, **15**−**17**, and **19** for 13 h, Shen and his co-workers observed the presence of a large number of big LC3 puncta in the HEK293-GFP-LC3 cells (Table 5). This observation of theirs was similar to the results obtained when the bafilomycin A1 group was used as the positive control. When the HEK293-GFP-LC3 cells were treated with 10 μM and 20 μM of compounds **15** and **16**, different levels of LC3 puncta were found to aggregate in all of the HEK293-GFP-LC3 cells. Subsequently, Shen et al. (2022) investigated the effects of compounds **15** and **16** on autophagy markers p62 and LC3-II. Their experiment demonstrated that the protein levels of LC3-II were upregulated after treating the HEK293-GFP-LC3 cells with 10 μM and 20 μM of compounds **15** and **16** (Table 5). p62, the selective autophagy substrate, simultaneously increased, thus indicating that compounds **15** and **16** could have inhibited the HEK293-GFP-LC3 cell autophagy as effectively as bafilomycin A1.

#### 4.2.4. Antibacterial and Anti-Quorum Sensing Activities

Park et al. (2017) reported the antibacterial activity of acylphenols through an *S. pneumoniae* sialidases inhibition assay (Table 6). Compounds **2** and **3,** respectively, showed strong pneumococcal sialidases inhibition for NanA (IC_50_ = 0.4 μM and 0.3 μM), NanB (IC_50_ = 5.7 μM and 3.6 μM), and NanC (IC_50_ = 14.3 μM and 2.9 μM). While compounds **2** and **3** inhibited the activity of NanA by the competitive inhibition mechanism, they, however, were noncompetitive inhibitors of NanB and NanC (Table 6). Since the inhibitory activities of compounds **2** and **3** against NanA and NanB were more potent compared to DANA (Neu5Ac2en), these results therefore suggested that both acylphenols could be potential agents for combating *S. pneumoniae* infection. 

**Table 6:** The antibacterial and anti-quorum sensing activities of acylphenols and dimeric acylphenols from the genus *Myristica*.

*Myristica* spp.	Part of the Plant Investigated	Acylphenols/Dimeric Acylphenols	Main Findings	Reference
*M. cinnamomea* King	Bark	Malabaricone A (**1**) Malabaricone B (**2**) Malabaricone C (**3**) Giganteone A (**20**)	**Anti-quorum sensing inhibitory activity against *E. coli* (pSB401) and *E. coli* (pSB1075) biosensors.** *E. coli* (pSB401) **1**–**3**: No significant bioluminescence inhibition **20**: Increase in concentration from 95 μg/mL to 380 μg/mL Showed significant inhibition of the bioluminescence *E. coli* (pSB1075) **1**–**3**: No significant bioluminescence inhibition **20**: Increase in concentration from 285 μg/mL to 380 μg/mL Showed significant inhibition of the bioluminescence	[36]
*M. fragrans* Houtt.	Seeds	Malabaricone B (**2**) Malabaricone C (**3**)	**Antibacterial activity: In vitro inhibitory activity against *Streptococcus pneumoniae* sialidases NanA, NanB and NanC.** NanA **2**: IC_50_ = 0.4 μM **3**: IC_50_ = 0.3 μM DANA (Neu5Ac2en) (positive control): IC_50_ = 4.8 ± 1.1 μM Inhibition mode (K_i_, μM) **2**: Competitive (0.5 ± 0.03 μM) **3**: Competitive (0.1 ± 0.01 μM) NanB **2**: IC_50_ = 5.7 μM **3**: IC_50_ = 3.6 μM DANA (Neu5Ac2en) (positive control): IC_50_ = 45.1 ± 2.5 μM Inhibition mode (K_i_, μM) **2**: Noncompetitive (5.6 ± 1.7 μM) **3**: Noncompetitive (3.0 ± 0.2 μM) NanC **2**: IC_50_ = 14.3 μM **3**: IC_50_ = 2.9 μM Inhibition mode (K_i_, μM) **2**: Noncompetitive (5.8 ± 0.2 μM) **3**: Noncompetitive (2.1 ± 0.05 μM)	[43]
*M. malabarica* Lam.	Seeds	Malabaricone C (**3**)	**Antibacterial activity: In vitro inhibitory activity against Gram-positive (*S. aureus*) and Gram-negative (*P. aeruginosa*) bacteria.** Test plate (1:2) ratio of smart multifunctional epoxy coating incorporated with bio-nanocomposites of **3**: No microbial colonies after 1 h incubation in saline meaning 99.99% killing Test plate with 0×, 10×, 100× dilution (1:2) ratio of smart multifunctional epoxy coating incorporated with bio-nanocomposites of **3**: No colonies present	[54]

Recently, Rajimol et al. (2022) demonstrated that compound **3** was an excellent antimicrobial agent in smart coating, showing 99.99% efficiency, and the coating exhibited activity against both gram-positive (*S*. *aureus*) and negative (*P*. *aeruginosa*) bacteria (Table 6). 

Sivasothy et al. (2016a) examined the quorum sensing inhibitory activity of acylphenols and dimeric acylphenols (Table 6). Compound **20** was identified as a potential anti-quorum sensing agent. Increasing the concentration of compound **20** was found to significantly inhibit the bioluminescence produced by both *E. coli* (pSB401) and *E. coli* (pSB1075), respectively (Table 6). Therefore, compound **20** has the potential to prohibit bacterial pathogenicity.

#### 4.2.5. Antidiabetic and Antiglycation Activities 

Sivasothy et al. (2016b) reported compound **21** to be a potent mixed-type α-glucosidase enzyme inhibitor with a 3-folds higher affinity towards the free enzymes (Table 7). Although the ability of compound **11** to inhibit the activity of the α-glucosidase enzyme was not as significant as that of compound **21**, nevertheless, the inhibitory potential of the former was four times greater compared to acarbose (Table 7). 

**Table 7:** The antidiabetic and antiglycation activities of acylphenols and dimeric acylphenols from the genus *Myristica*.

*Myristica* spp.	Part of the Plant Investigated	Acylphenols/Dimeric Acylphenols	Main Findings	Reference
*M. cinnamomea* King	Bark	Cinnamomeone A (**11**) Giganteone D (**21**)	**Antidiabetic activity: In vitro α-Glucosidase enzyme inhibitory activity** **11**: IC_50_ = 358.80 μM **21**: IC_50_ = 5.05 μM Acarbose (positive control): IC_50_ = 1449.67 μM Lineweaver–Burk plot analysis for **21**: Mode of inhibition: mixed-type Ki_1_: 22.16 μM Ki_2_: 72.49 μM	[37]
*M. cinnamomea* King	Bark	Malabaricone A (**1**) Malabaricone B (**2**) Malabaricone C (**3**) Malabaricone E (**5**) Giganteone A (**20**)	**Antidiabetic activity: In vitro α-Glucosidase enzyme inhibitory activity** **1**: IC_50_ = 236.03 μM **2**: IC_50_ = 210.63 μM **3**: IC_50_ = 59.61 μM **5**: IC_50_ = 35.86 μM **20**: IC_50_ = 39.52 μM Acarbose (positive control): IC_50_ = 1449.67 μM	[61]
*M. fatua* Houtt.	Seeds	Promalabaricone B (**7**)	**Antidiabetic activity: In vitro α-Amylase and α-Glucosidase enzymes inhibitory activities** α-amylase enzyme inhibitory activity **7**: IC_50_ = 82.00 ± 1.23 μM Acarbose (positive control): IC_50_ = 8.20 ± 1.23 μM (*p* < 0.01). α-glucosidase enzyme inhibitory activity **7**: IC_50_ = 32.70 ± 0.47 μM (*p* < 0.01) Acarbose (positive control): IC_50_ = 52.04 ± 0.9 μM **Antiglycation activity** **7**: IC_50_ = 227.26 ± 0.80 μM (*p* < 0.01) Ascorbic acid (positive control): IC_50_ = 155.38 ± 0.55 μM (*p* < 0.01) **7**: Glucose uptake [46.3% (2.5 μM)] Metformin (positive control): 35.2% of glucose uptake at 100 μM under identical experimental conditions	[40]
*M. fatua* Houtt. var. *magnifica* (Bedd.) Sinclair	Bark	Malabaricone A (**1**) Malabaricone B (**2**) Malabaricone C (**3**) 1-(2-hydroxy-6-methoxyphenyl)-9-(4-hydroxyphenyl)nonan-1-one (**6**) 1-(2,6-dihydroxyphenyl)tetradecan-1-one (**9**) 1-(2-hydroxy-6-methoxyphenyl)tetradecan-1-one (**10**)	**Antidiabetic activity: In vitro α-Amylase and α-Glucosidase enzymes inhibitory activities ** α-amylase enzyme inhibitory activity **1**: IC_50_ = 19.07 ± 0.517 μM **2**: IC_50_ = 12.89 ± 0.068 μM **3**: IC_50_ = 10.63 ± 0.171 μM **6**: IC_50_ = 32.27 ± 0.500 μM **9**: IC_50_ = 74.12 ± 1.278 μM **10**: IC_50_ = 39.01 ± 1.20 μM Acarbose (positive control): IC_50_ = 8.93 ± 0.48 μM α-glucosidase enzyme inhibitory activity **1**: IC_50_ = 91.44 ± 1.245 μM **2**: IC_50_ = 63.70 ± 0.546 μM **3**: IC_50_ = 43.61 ± 0.620 μM **6**: IC_50_ = 94.53 ± 0.875 μM **9**: IC_50_ = 171.90 ± 0.890 μM **10**: IC_50_ = 256.71 ± 0.492 μM Acarbose (positive control): IC_50_ = 66.57 ± 0.982 μM **Antiglycation activity** AGEs inhibitory activity **1**: IC_50_ = 19.28 ± 0.0454 μM **2**: IC_50_ = 40.34 ± 0.0948 μM **3**: IC_50_ = 14.99 ± 0.114 μM **6**: IC_50_ = 104.27 ± 0.933 μM **9**: IC_50_ = 120.84 ± 0.547 μM **10**: IC_50_ = 192.09 ± 0.915 μM Ascorbic acid (positive control): IC_50_ = 155.38 ± 0.547 μM **2**: Glucose uptake [37.5% (10 μM), 45.8% (25 μM), 52.7% (50 μM)] Metformin (positive control): 36.6% of glucose uptake at 100 μM under identical experimental conditions	[41]
*M. fragrans* Houtt.	Seeds	Malabaricone C (**3**)	**Antidiabetic activity: In vitro α-Glucosidase enzyme inhibitory activity** **3**: IC_50_ = 20.97 ± 0.17 μg/mL	[10]

Sivasothy et al. (2022) later identified compounds **1**, **2**, **3**, **5,** and **20** to be more effective in inhibiting the activity of the α-glucosidase enzyme compared to acarbose. Compounds **3** and **5** were stronger α-glucosidase enzyme inhibitors than compounds **1** and **2** (Table 7). Sivasothy et al. (2022) deduced that the higher number of hydroxyl groups in the structure of the former two acylphenols could have resulted in its lower IC_50_ values compared to the latter two acylphenols (Figure 1). Sivasothy et al. (2022) further postulated that the inhibiting potential of compound **20,** which was almost double that of compound **3,** could have resulted from the dimeric acylphenol possessing double the number of hydroxyl groups compared to its monomeric acylphenol (Figure 1 and Figure 2). 

Based on the findings by Prabha et al. (2018), compounds **2** and **3** were determined to be more effective α-glucosidase enzyme inhibitors compared to acarbose (Table 7). Though compounds **2** and **3** exhibited a slightly weaker potential in inhibiting the activity of the α-amylase enzyme compared to acarbose, their IC_50_ values were comparable to that of acarbose. Compound **1** on the other hand can only be considered a moderate α-amylase enzyme inhibitor compared to acarbose (Table 7). When the α-glucosidase and α-amylase enzyme inhibitory activities of compounds **1**–**3** and **6** were compared to those of compounds **9** and **10**, the weaker activities of compounds **9** and **10** enabled the researchers to deduce that the presence of two aromatic rings with hydroxyl groups bonded to them was a prerequisite for the respective activities (Figure 1). Prabha et al. (2018) also concluded that the presence of a methoxyl group at position C-21 in compound **6** could have decreased its α-amylase enzyme inhibitory activity compared to compound **1,** whose corresponding position bore a hydroxyl group instead (Figure 1). According to Prabha et al. (2018), compounds **1**–**3**, **6,** and **9** also displayed antiglycation properties (Table 7). These five acylphenols effectively inhibited the formation of AGEs compared to ascorbic acid. Compounds **1**–**3** were 2.5 to 8 folds more potent in inhibiting the formation of AGEs compared to compounds **6** and **9**. Though all of the compounds at concentrations of 10, 25, and 50 μM exhibited moderate to good glucose uptake in a dose dependent manner, only compound **2** demonstrated a significantly better stimulation of glucose uptake as compared to metformin under identical experimental conditions (Table 7).

Prabha et al. (2021) revealed compound **7** to possess an α-glucosidase enzyme inhibitory activity which was 2.5 folds stronger compared to its potential in inhibiting the activity of the α-amylase enzyme. When compared to acarbose, compound **7** can only be regarded as a strong α-glucosidase enzyme inhibitor (Table 7). Prabha et al. (2021) also considered compound **7** to possess moderate antiglycation effects compared to ascorbic acid and a greater potential compared to metformin in increasing glucose uptake in cells under identical operating conditions. Furthermore, compound **7** was also able to enhance the translocation and expression of GLUT4 and upregulate the AMPK pathway (Table 7).

According to Li et al. (2020), compound **3** inhibited the activity of the α-glucosidase enzyme in a dose dependent manner with an IC_50_ value of 20.97 ± 0.17 μg/mL (Table 7). The inhibition of the enzyme increased from around 15% to around 90% when the concentration of compound **3** was increased from 0.6 μg/mL to 2.2 μg/mL.

#### 4.2.6. Anti-Alzheimer’s Disease Activity

Cuong et al. (2014) demonstrated that compound **3** had a weak AChE inhibitory activity compared to the positive control Berberine (Table 8). On the other hand, Sathya et al. (2020) described that compound **3** was found to show potent AChE inhibitory activity with an IC_50_ value of 2.06 ± 0.04 μg/mL (Table 8). 

**Table 8:** The anti-Alzheimer’s Disease activity of acylphenols and dimeric acylphenols from the genus *Myristica*.

*Myristica* spp.	Part of the Plant Investigated	Acylphenols/Dimeric Acylphenols	Main Findings	Reference
*M. cinnamomea* King	Fruits	Malabaricone A (**1**) Malabaricone B (**2**) Malabaricone C (**3**) Malabaricone E (**5**) Maingayone A (**24**) Maingayone B (**25**)	**Anti-Alzheimer’s Disease activity: In vitro cholinesterase enzymes [(AChE) and (BChE)] inhibitory activities** AChE **1**: IC_50_ = 1.31 ± 0.17 μM **2**: IC_50_ = 1.84 ± 0.19 μM **3**: IC_50_ = 1.94 ± 0.27 μM **5**: IC_50_ = 6.44 ± 0.85 μM **24**: IC_50_ = 12.66 ± 1.48 μM **25**: IC_50_ = 30.67 ± 8.14 μM Physostigmine (positive control): IC_50_ = 0.08 ± 0.02 μM BChE **1**: IC_50_ = 39.21 ± 3.46 μM **2**: IC_50_ = 1.76 ± 0.21 μM **3**: IC_50_ = 2.80 ± 0.49 μM **5**: IC_50_ = 6.65 ± 0.13 μM **24**: IC_50_ = 10.51 ± 2.07 μM **25**: IC_50_ = 12.52 ± 2.86 μM Physostigmine (positive control): IC_50_ = 0.22 ± 0.02 μM K_iAChE_ and K_iBChE_ **2**: 4.33 μM and 0.56 μM **3**: 5.86 μM and 11.46 μM	[38]
*M. fragrans* Houtt.	Aril	Malabaricone C (**3**)	**Anti-Alzheimer’s Disease activity: In vitro AChE inhibitory activity** **3**: IC_50_ = 2.06 ± 0.04 μg/mL Donepezil HCI (positive control): IC_50_ = 0.03 ± 0.00 μg/mL	[44]
*M. fragrans* Houtt.	Seeds	Malabaricone C (**3**)	**Anti-Alzheimer’s Disease activity: In vitro AChE inhibitory activity** **3**: IC_50_ = 44.0 μM Berberine (positive control): IC_50_ = 0.1 μM	[42]
*M. fragrans* Houtt.	Aril	Malabaricone A (**1**) Malabaricone C (**3**)	**Anti-Alzheimer’s Disease activity: In vitro AChE and BChE inhibitory activities** AChE **1**: IC_50_ = 67.41 ± 1.52 μM **3**: IC_50_ = 25.02 ± 0.95 μM Donepezil (positive control): IC_50_ = 0.07 ± 0.00 μM BChE **1**: IC_50_ = 27.16 ± 0.06 μM **3**: IC_50_ = 22.36 ± 0.03 μM Donepezil (positive control): IC_50_ = 4.73 ± 0.91 μM K_iAChE_ and K_iBChE_ **3**: 25.01 μM and 22.36 μM	[46]

Abdul Wahab et al. (2016) reported that the acylphenols (**1**–**3** and **5**) and the dimeric acylphenols (**24** and **25**) isolated and characterized from the fruits of *M. cinnamomea* had the potential to treat Alzheimer’s Disease via the inhibition of the activities of the cholinesterase enzymes; AChE and BChE (Table 8). Compounds **2** and **3** were identified as strong mixed mode dual inhibitors, with almost equal AChE and BChE inhibiting potentials. Compound **5,** though it was also identified as a dual inhibitor, was only moderate in its ability to inhibit the activities of both the enzymes. Compound **1,** on the other hand, was an AChE selective inhibitor. As for compounds **24** and **25**, they were moderate AChE and BChE inhibitors (Table 8). The researchers concluded that the AChE inhibiting potential of compounds **1**–**3** may have decreased with the increase in the number of hydroxyl groups in their ring b (Figure 1). The lower AChE inhibiting potential of compound **5** upon comparison to compound **2** could have resulted from the additional hydroxyl group in its ring a (Figure 1). With regard to the BChE inhibitory activity, compound **1** showed the weakest activity, which could have been due to the absence of hydroxyl groups in its ring b compared to compounds **2**, **3,** and **5,** which bore one or two hydroxyl groups in their ring b (Figure 1). The researchers further deduced that dimerization, which in turn resulted in the bulkiness of compounds **24 and 25,** could have contributed to the decrease in their activities compared to their monomers (compounds **2** and **3**) (Figure 1 and Figure 2). Rastegari et al. (2022) in their investigation revealed that compounds **1** and **3** had the potential in inhibiting the activities of the AChE and BChE. While compound **3** was identified as a non-competitive dual AChE and BChE inhibitor with almost equal enzyme-inhibiting strength, compound **1** was a more effective inhibitor of the activity of the BChE compared to the activity of the AChE (Table 8).

#### 4.2.7. Anti-Allergic Activity

According to Morikawa et al. (2018), compound **3** inhibited the release of β-hexosaminidase, a biomarker of the anti-allergic activity through antigen-IgE-stimulated degranulation in rat basophilic leukemia cells (RBL-2H3) (Table 9). The inhibitory activity of compound **3** was greater than that of the antiallergic medicines: tranilast and ketotifen fumalate. Compound **3** also inhibited the production of the antigen-stimulated tumor necrosis factor-α, an important process in the late phase of type I allergic reactions (Table 9).

**Table 9:** The anti-allergic activity of acylphenols from the genus *Myristica*.

*Myristica* spp.	Part of the Plant Investigated	Acylphenols	Main Findings	Reference
*M. fragrans* Houtt.	Aril	Malabaricone C (**3**)	**Anti-allergic activity** The in vitro inhibition of the release of β-hexosaminidase in RBL-2H3 cells **3**: IC_50_ = 20.7 μM Tranilast (positive control): IC_50_ = 282 μM Ketotifen fumalate (positive control): IC_50_ = 158 μM Inhibition of the production of antigen-stimulated tumor necrosis factor-α **3**: IC_50_= 39.5 μM	[7]

#### 4.2.8. Anti-Anxiety Activity

Modulatory effects in the cannabinoid system through the inhibition of the activities of the fatty acid amide hydrolase (FAAH) and the monoacylglycerol lipase (MAGL) enzymes may serve as therapeutic potentials in treating disorders such as mood and anxiety [11]. El-Alfy et al. (2019) demonstrated that compound **3** was only able to inhibit the activity of the FAAH enzyme, though not as effectively as JZL 195 (Table 10). 

**Table 10:** The anti-anxiety activity of acylphenols from the genus *Myristica*.

*Myristica* spp.	Part of the Plant Investigated	Acylphenols	Main Findings	Reference
*M. fragrans* Houtt.	Kernel	Malabaricone B (**2**) Malabaricone C (**3**)	**Anti-anxiety activity: In vitro inhibitory activity of the endocannabinoid system through the inhibition of the fatty acid amide hydrolase (FAAH) and the monoacylglycerol lipase (MAGL) enzymes** FAAH enzyme **2**: No inhibition **3**: IC_50_ = 38.29 ± 6.18 μM JZL 195 (positive control): 0.045 ± 0.002 μM MAGL enzyme **2**: No inhibition **3**: No significant inhibition JZL 195 (positive control): 0.71 ± 0.31 μM	[11]

#### 4.2.9. Antihypertensive Activity

Rathee et al. (2016) reported that the chronic oral administration of compound **3**, a natural antioxidant, could reduce blood pressure (BP) and attenuate cardiovascular remodeling in deoxycorticosterone acetate (DOCA)-salt hypertensive rats (Table 11). DOCA-salt hypertensive rats had significantly high systolic BP (SBP), which was related to organ hypertrophy, collagen depositions, inflammatory infiltrations in cardiac and aortic sections, reduction in plasma, total antioxidant status and NO level, and increase in TBARS, PGI2, and vasoconstrictors (AVP, Big ET, and ET-1). DOCA-salt also decreased vascular relaxation caused by smooth muscles and endothelium in rats. Compound **3** reversed all these changes in the DOCA-salt hypertensive rats and improved their vascular reactivity. Compound **3** was found to exert anti-hypertensive properties in DOCA-salt hypertensive rats by reducing oxidative stress and organ hypertrophy and by improving endothelial and vascular functions (Table 11).

**Table 11:** The antihypertensive activity of acylphenols from the genus *Myristica*.

*Myristica* spp.	Part of the Plant Investigated	Acylphenols	Main Findings	Reference
*M. malabarica* Lam.	Rind	Malabaricone C (**3**)	**Anti-hypertensive activity** **3**: Lowers systolic BP (SBP) of the DOCA-salt hypertensive rats without restoring it to control level, reduces organ hypertrophy, reduces oxidative stress (OS), reduces vasoconstriction, reduces ventricular and vascular collagen deposition and inflammation, improves vascular, endothelial, and smooth muscle dysfunction in DOCA-salt hypertensive rats.	[52]

#### 4.2.10. Anti-Obesity Activity

Sphingomyelin synthase (SMS) is a membrane protein family that includes two isoforms: sphingomyelin synthase 1 (SMS1) and sphingomyelin synthase 2 (SMS2) [62]. The increase in SM and DAG which is produced by the SMSs will lead to obesity and insulin resistance [63,64]. Othman et al. (2019) reported that compounds **1**–**3** and **5** exhibited strong inhibitory activities against SMS 1 and SMS 2 (Table 12). Compound **3** was highly efficacious in preventing oleic acid uptake across the membrane, by reducing lipid droplet formation in vitro. The potential of compound **3** against diet-induced obesity and lipid metabolism in vivo was also investigated. Compound **3** was found to reduce body weight gain, improve glucose tolerance, and decrease lipid accumulation in the liver in vivo.

**Table 12:** The anti-obesity activity of acylphenols from the genus *Myristica*.

*Myristica* spp.	Part of the Plant Investigated	Acylphenols	Main Findings	Reference
*M. cinnamomea* King	Fruits	Malabaricone A (**1**) Malabaricone B (**2**) Malabaricone C (**3**) Malabaricone E (**5**)	**Anti-obesity activity: In vitro and in vivo Sphingomyelin Synthases (SMSs) 1 and 2 enzymes inhibitory activities** SMS 1 enzyme **1**: IC_50_ = 4 μM **2**: IC_50_ = 3.5 μM **3**: IC_50_ = 3 μM **5**: IC_50_ = 6 μM SMS 2 enzyme **1**: IC_50_ = 4 μM **2**: IC_50_ = 2.5 μM **3**: IC_50_ = 1.5 μM **5**: IC_50_ = 4.5 μM Compound **3** was highly efficacious in preventing oleic acid uptake across the membrane, which, in turn, reduced lipid droplet formation in vitro. Compound **3** was able to reduce body weight gain, improve glucose tolerance, and decrease lipid accumulation in the liver in vivo.	[65]

#### 4.2.11. Anti-Dengue Activity

According to Sivasothy et al. (2021), compounds **3** and **5** demonstrated antiviral activity against the DENV-2 NS2B/NS3 protease (Table 13). Compounds **3** and **5** both inhibited the activity of the DENV-2 NS2B/NS3 protease, with the latter being the stronger inhibitor among the two and around 1.4 times more potent than that of quercetin itself.

**Table 13:** The anti-dengue activity of acylphenols from the genus *Myristica*.

*Myristica* spp.	Part of the Plant Investigated	Acylphenols	Main Findings	Reference
*M. cinnamomea* King	Fruits	Malabaricone A (**1**) Malabaricone B (**2**) Malabaricone C (**3**) Malabaricone E (**5**)	**Anti-dengue activity: In vitro DENV-2 NS2B/NS3 protease inhibitory activity** **1**–**2**: less than 70% inhibition at 200 μg/mL. **3**: IC_50_ = 27.33 ± 5.45 μM **5**: IC_50_ = 7.55 ± 1.64 μM Quercetin (positive control): IC_50_ = 10.48 ± 2.14 μM	[66]

## 5. Conclusions

The results from this review were summarized and synthesized from a pool of 35 individual studies that investigated the acylphenol and dimeric acylphenol composition present in the fruits, leaves, and bark of eight different species within the genus *Myristica,* along with their in vitro and in vivo pharmacological properties. This review provides a comprehensive overview of the existing research from 2013 to 2022. Thus, the results summarized within this review provide a broad up to date representation of the pharmacological activities of this class of secondary metabolites. The review, therefore, provides scientific evidence that the medicinal properties of the genus *Myristica* could have been attributed to the bioactive acylphenols and dimeric acylphenols present in various parts of its plants. Furthermore, this review also highlights the potential for the development of the acylphenols and dimeric acylphenols from the genus *Myristica* as pharmaceutical products. Nevertheless, additional research on their pharmacokinetics, metabolism, side effects, and toxicity is needed to show the effectiveness and safety of these compounds.

## Data Availability

Not applicable.

## List of Abbreviations

ABTS	2,2′-azino-bis(3-ethylbenzothiazoline-6-sulfonic acid)
AChE	Acetylcholinesterase enzyme
AGEs	Advanced glycated end products
AMPK	AMP-activated protein kinases
ASK1	Apoptosis stimulating kinase 1
BAX	Bcl-2 Associated X-protein
BChE	Butrylcholinesterase enzyme
BCL-2	B-cell lymphoma-2
BCL-XL	B-cell lymphoma-extra large
BHT	Butylhydroxytoluene
BSO	Buthionine sulfoximine
CCRF CEM	Human leukemic lymphoblasts
CEM/ADR5000	Multidrug-resistant leukemia cells
COX-2	Cyclooxygenase 2
DAG	Diacylglycerol
DANA (Neu5Ac2en)	2-Deoxy-2,3-dehydro-n-acetyl-neuraminic acid
DENV-2	Dengue virus 2
DNA	Deoxyribonucleic acid
DPPH	2,2-Diphenyl-1-picryl-hydrazyl-hydrate
*E. coli*	Escherichia coli
GI	Gastrointestinal
GLUT4	Glucose transporter type 4
HPLC	High performance liquid chromatography
IL	Interleukin
JNK	c-Jun N-terminal kinase
JZL 195	(4-nitrophenyl) 4-[(3-phenoxyphenyl)methyl]piperazine-1-carboxylate
Krt1	Keratin 1
LDL	Low-density lipoprotein
LT	Leukotriene
LTB_4_	Leukotriene B4
LOX	Lipoxygenase
mTOR	Mammalian target of rapamycin
MTT	2,5-diphenyl-2H-tetrazolium bromide
NAC	N-acetyl-l-cysteine
NO	Nitric oxide
RBL	Rat basophilic leukemia
PEG-SOD	Superoxide dismutase–polyethylene glycol
PGI2	Prostaglandin I2
ROS	Reactive oxygen species
S100A9	S100 calcium binding protein A9
shRNA	Short hairpin RNA
SM	Sphingomyelin
TBARS	Thiobarbituric acid reactive substance
TLC	Thin-layer chromatography
